# A randomized controlled trial comparing parent child interaction therapy - toddler, circle of security– parenting™ and waitlist controls in the treatment of disruptive behaviors for children aged 14–24 months: study protocol

**DOI:** 10.1186/s40359-020-00457-7

**Published:** 2020-08-31

**Authors:** Jane Kohlhoff, Sara Cibralic, Nancy Wallace, Susan Morgan, Cathy McMahon, Erinn Hawkins, Valsamma Eapen, Nancy Briggs, Anna Huber, Cheryl McNeil

**Affiliations:** 1 School of Psychiatry, University of New South Wales, P.O. Box 241, Villawood, NSW 2163 Australia; 2 Karitane, Carramar, NSW Australia; 3 Ingham Institute for Medical Research, Liverpool, NSW Australia; 4 Macquarie University, Ryde, NSW Australia; 5 Griffith University, Gold Coast, QLD Australia; 6 South Western Sydney Local Health District, Liverpool, Australia; 7 Mark Wainwright Analytical Centre, University of New South Wales, Kensington, NSW Australia; 8 Families In Mind Psychology, Canberra, Australia; 9 West Virginia University, Morgantown, WV USA

**Keywords:** Toddlers, Parent-child interaction therapy – toddlers, Circle of security, Attachment, Disruptive behaviors, Emotion regulation

## Abstract

**Background:**

It is common for toddlers to display disruptive behaviors (e.g., tantrums, aggression, irritability) but when these become severe and persistent they can be the start of a trajectory towards poor outcomes in childhood and adolescence. Parent Child Interaction Therapy - Toddler is an intervention model designed to meet the specific developmental needs of toddlers aged 12–24 months presenting with disruptive behaviors.

**Methods:**

This study will use a randomized controlled design to evaluate the efficacy of the Parent Child Interaction Therapy - Toddler intervention for children aged 14–24 months with disruptive behaviors. Ninety toddlers with parent-reported disruptive behavior will be randomly allocated to either Parent Child Interaction Therapy - Toddler, Circle of Security– Parenting™ or a waitlist control group. Key parenting capacity outcome variables will include positive and negative parenting, parenting sensitivity, parental sense of competence in managing negative toddler emotions, parent sense of caregiving helplessness, parent mentalizing about the child, parent emotion regulation, child abuse potential and parental stress. Key outcome variables for children will include child social-emotional functioning (initiative, relationship functioning, self-regulation), child emotion regulation, child attachment security, and child behavior.

**Discussion:**

Delivered in the early intervention period of toddlerhood, Parent Child Interaction Therapy - Toddler has the potential to bring about significant and lasting changes for children presenting with early onset behavioral issues.

**Trial registration:**

Australian New Zealand Clinical Trials Registry (ANZCTR), 12618001554257. Registered 24 September 2018 – retrospectively registered.

## Background

Behavioral difficulties in infancy and toddlerhood including persistent tantrums or aggression can signify the start of a trajectory towards poor psychiatric and psychosocial problems across the lifespan [1]. While such behaviors typically represent normal responses to the challenges of the developmental stage and may resolve naturally, evidence suggests that persistent and untreated early onset behavioral difficulties can predict social-emotional challenges, academic problems and conduct disorders in middle childhood and adolescence [2, 3], and psychopathology and anti-social behavior in adulthood [4, 5]. The wider impacts in terms of parental stress [6], added risk for harsh and abusive parenting [7], and social concerns [8], also are also significant and well established.

Although various genetic, social, and temperament pathways to early onset behavioral difficulties have been proposed [9, 10], it is widely agreed that the quality of the early parent-child attachment relationship plays a key role in determining the child’s capacity for behavioral and emotional control [11]. Attachment theory and supporting empirical evidence [12, 13] suggest that when children in the early years of life receive ‘sensitive’ caregiving (defined as a parent’s capacity to notice and correctly interpret the child’s signals, and then to respond appropriately and promptly [14]) - they are more likely to develop a ‘secure’ attachment relationship with the caregiver. Behaviorally, this manifests in the Strange Situation Procedure (SSP) [15] - the well-known experimental paradigm designed to assess attachment patterns in infants - as distress on separation from the caregiver followed by active utilisation of the caregiver on reunion for emotional support. It is thought to be within the context of a safe and supportive attachment relationship that the securely attached child develops the social-emotional skills required for behavioral and emotional control [11], hallmarks of healthy psychological development. In contrast, children who have received insensitive, unresponsive or inconsistent caregiving in their early years tend to develop an alternative strategy to organise their behavior, described as an ‘insecure’ attachment pattern (either avoidance or ambivalence) [15]. Finally, a ‘disorganized’ attachment pattern can also be observed among children who have received parenting with frightened, frightening or dissociative features [16, 17]. Typically observed in the SSP as odd, contradictory, disorganized behaviors in the presence of the parent, this pattern is thought to represent a breakdown or incoherence in the child’s organisational strategy and is known to be associated with the poorest psychological outcomes [17–20]. These theoretical assertions are backed by empirical evidence showing that insecure and disorganized attachment patterns in infancy predict disruptive/externalizing behaviors in later childhood [21].

In seeking to better understand the intergenerational transmission of caregiving quality and attachment, research attention has naturally turned to the investigation of parental factors and characteristics that are associated with, and predictive of, parenting sensitivity and infant attachment security. A parent’s own state-of-mind regarding his or her own attachment history has been identified as one of the strongest predictors of both parental sensitivity and child attachment security [22, 23]; however, other predictors have also been identified. These include parents’ capacity to regulate thier own emotions [24, 25] and their “mentalizing” ability - their ability to keep the child’s ‘mind’ (i.e., thoughts, feelings, intentions, desires) in his or her mind [26–28].

Given the opportunities that early childhood presents for intervention to support and enhance parent-child relationships, a number of attachment-based parenting intervention approaches targeting high-risk children and caregivers have been developed [29]. Examples include Video-feedback Intervention to promote Positive Parenting and Sensitive Discipline (VIPP-SD) [30], Attachment and Biobehavioral Catchup (ABC) [31, 32], and the Circle of Security Intensive Intervention (COS-I) [33]. Each of these interventions aims to address parenting sensitivity and some explicitly target infant attachment and disruptive/externalizing child behaviors. Evidence for the efficacy of these programs in relation to the specific age of toddlerhood (12–24 months) and for reducing disruptive child behaviors, however, is limited and equivocal. VIPP-SD [30] involves review of video-taped parent-child interaction sessions in the home and aims to increase parental sensitivity and sensitive parental discipline, defined as the ability to take into account the child’s perspective and signals when discipline is required. VIPP-SD has been shown to be effective in enhancing maternal attitudes toward sensitivity and sensitive discipline and in promoting sensitive discipline interactions. The intervention also resulted in decreased overactive problem behaviors, but only among children in families with elevated levels of marital discord and daily hassles (e.g., money problems or troubles at work); child externalizing behaviors remained unchanged. ABC is based around active coaching of the parent from a therapist in ten 1-h home-based sessions, with coaching focused specifically on enhancing nurturance of the child, following the child’s lead with delight, and reducing frightening/intrusive behavior. ABC has been shown to be associated with a range of positive outcomes including decreased rates of disorganized attachment and increased rates of secure attachment [34], normalized diurnal cortisol production in children [35], enhanced child executive functioning [36], and improved emotion expression [37]. The efficacy of the program for disruptive or externalizing behavior in the toddler age group, however, has not been specifically demonstrated. COS-I comprises a mix of individual and group components spread over 20 weeks, and aims to increase caregiver sensitivity and responsiveness to child cues, empathy for the child by supporting parental reflective functioning, recognition and understanding of child attachment cues, and awareness of the impact of the caregiver’s own attachment history on caregiving. COS-I has not yet been evaluated using a RCT but open trial designs suggest positive changes in terms of child attachment security, internalizing and externalizing symptoms, and in parenting stress and parent mood problems [38–41]. Given the wide age range in these studies (1–8 years), specific outcomes for toddlers aged less than 2 years are unknown. Taken together, it is clear that there is a need for new intervention models to address the unique needs of parents and toddlers in the 12–24 age range experiencing early onset disruptive or externalizing behaviors.

‘Parent Child Interaction Therapy – Toddler’ (PCIT-T) [42] is a new parenting intervention program designed to address behavioral problems in young toddlers aged 12–24 months. Drawing on both attachment and social learning theories, and grounded in developmental principles, PCIT-T assumes that (i) problematic behaviors in young toddlers indicate emotional dysregulation rather than deliberate, intentional attention-seeking acts or components of a coercive parent-child interaction cycle, (ii) the early parent-child attachment relationship is the vehicle through which capacities for emotion and behavior regulation in the young child emerge and are consolidated, and (iii) young toddlers have the capacity to learn new skills. The program aims to improve basic parenting skills (e.g., increase use of praise and reduce negative language directed at the child), to increase the parent’s awareness of, and sensitivity to, the child’s emotional experience and needs, to improve the parent’s capacity to support the child’s emotional regulation, to improve the parent’s own emotional regulation skills, and to improve the child’s listening skills and compliance. Through improvements in these domains, it is expected that infant attachment security, child social emotional functioning including emotion regulation, and externalizing behaviors, will be natural flow-on effects. PCIT-T is an adaptation of standard PCIT [43], developed for children aged 2–7 years presenting with conduct problems. Full descriptions of standard PCIT and overviews of the evidence base can be found elsewhere [43, 44]. Although there are some shared features between standard PCIT and PCIT-T, PCIT-T differs significantly from standard PCIT in its explicit emphasis on emotion regulation for both child and parent, and lack of a formal ‘discipline’ phase or time out procedure.

As is the case in standard PCIT, the PCIT-T program utilizes in-clinic parent-child play sessions with direct live coaching from the therapist, usually from behind a one-way mirror using a bluetooth in-ear microphone. There are two phases of the program, ‘Child Directed Interaction - Toddlers’ (CDI-T) and ‘Parent Directed Interaction-Toddlers’ (PDI-T). The CDI-T phase teaches parents to (a) use ‘P-R-I-D-E’ skills (labeled Praise, Reflections of child verbalizations, Imitating child play, Behavioral Descriptions, and Enjoyment); (b) reduce ‘Don’t behaviors’ (avoiding questions, commands and criticisms); and (c) use ‘C-A-R-E-S’ techniques to help the child regulate emotions when needed (Coming in close, Assisting the child, Reassuring the child, Labeling the Emotion, Soothing the child). In the CDI-T phase the parent is also taught to use “under-reaction and re-direction” in response to disruptive child behavior judged to be attention seeking (e.g., throwing toys) and to use a brief, developmentally appropriate limit-setting sequence in response to child aggression. The limit-setting sequence involves the parent coming close to the child, taking his or her hands, and, with direct eye contact, saying, “no hurting”. The parent then looks away for 3 s, and then looks back at the child and says, “no hurting, gentle hands” before rotating the child towards the toys and using PRIDE skills (and CARES skills, where needed) to re-engage the child in positive play. The PDI-T phase aims to promote toddler listening skills through a fun, game-like, guided compliance teaching sequence. Throughout both phases, to enhance parents’ ability to effectively implement the CDI-T and PDI-T skills, parents are also encouraged to develop their own emotion regulation skills through application of a parallel adult-focused C-A-R-E-S model [Coming in close to your own feelings, Assisting yourself to manage (e.g., deep breathing, progressive muscle relaxation), Reassuring yourself (cognitive challenging), Labeling Emotions experienced, Soothing yourself (e.g., self care)]. Between PCIT-T sessions, parents are encouraged to practice the skills with the child for 5 min on a daily basis. All sessions begin with a brief “check in” conversation in which the therapist and parent discuss and problem-solve any issues around home practice, and reflect on the parent’s experiences using the PRIDE and CARES skills (for both themselves and the child) during the week. Following the “check in”, the therapist observes the parent and child for 5 min (without providing coaching), and in this time codes the parent’s use of the PCIT-T skills (e.g., PRIDE skills, Don’t behaviors, CARES techniques). This allows the therapist to choose a focus area for the session and to track progress across sessions. The PCIT-T program is mastery-based, meaning that parent-child dyads progress from the CDI-T phase to the PDI-T phase when they show satisfactory use of the CDI-T skills in the 5 min observational period (i.e., they are observed to use 10 labeled praises, 10 behavioral descriptions and 10 reflections, and to adequately implement the CARES skills when needed). Graduation from the PDI-T phase takes place when the parent can competently implement the guided compliance teaching sequence. For most dyads, when PCIT-T is delivered twice weekly (30–40 min per session), CDI-T and PDI-T mastery is typically achieved after approximately 10–12 sessions.

Preliminary evidence to support the efficacy of PCIT-T has been positive. Kohlhoff and Morgan [45] initially made adaptations to the standard PCIT protocol including greater emphasis on preventing problem child behavior by increasing focus on the creation of safe, developmentally appropriate environments, and early intervention in the case of emotion dysregulation. There was no PDI phase but some developmentally appropriate limit setting was integrated into the CDI phase. Redirection was used as a primary form of behavior management, and parent-focused psychoeducation of developmentally appropriate expectations was infused throughout the treatment. Although the behavioral parent-training lens primarily used in PCIT remained at the core of many PCIT-T skills, an attachment-based emphasis on meeting the emotional and developmental needs of toddlers was a key focus of the treatment. Kohlhoff and Morgan [45] retrospectively reviewed outcomes of the first 29 cases treated with this early version of PCIT-T and showed that toddlers who received PCIT-T showed statistically and clinically significant improvements in child behavior and that their parents reported feeling less depressed. Kohlhoff and Morgan [46] subsequently reported results from a study in which mother-child dyads (*n* = 66) participated in a randomized controlled trial (RCT) study to evaluate outcomes of the CDI-T phase of the PCIT-T intervention versus a wait-list controlled condition. Results showed that parent-child dyads who received PCIT-T showed significantly greater gains in terms of increases in positive parenting skills, decreases in negative parenting behaviors, enhanced emotional availability including sensitivity, and reduced child externalizing behavior. When the sample was followed up 4-months after the completion of PCIT-T treatment, parent and child gains after PCIT-T were maintained [47]. The sample size at follow-up was small for categorical analyses, but there was a pattern of positive change from disorganized to organized infant attachment. A sub-sample of study participants also took part in a qualitative interview following treatment completion, and results indicated a high level of consumer satisfaction with the program [48].

The PCIT-T outcome research to date has been limited by the lack of a comparison intervention, no control group at follow-up and the limited range of outcome measures utilized (e.g. no measures of emotion regulation or parental representations) to enable greater understanding of the processes through which child behavior are achieved. There has also been no formal evaluation of the most recent version of the treatment model as articulated by Girard, Wallace, Kohlhoff, Morgan and McNeil [42], which includes structured handouts and record forms for parents, formalized implementation of the adult and child ‘CARES’ models for emotion regulation, and addition of the PDI-T phase for promotion of toddler listening skills and parental provision of effective commands.

### Study aims and hypotheses

The objective of the current study is to evaluate the most recent iteration of the PCIT-T model, as articulated by Girard and colleagues [42]. Study aims will be to 1) examine whether the PCIT-T intervention leads to positive changes in parenting capacity for parents of young toddlers presenting with early disruptive behaviors; and 2) examine social-emotional, attachment and behavioral outcomes for children who participate in PCIT-T.

Three conditions will be tested: a waitlist control group who are not treated, PCIT-T and a group receiving Circle of Security – Parenting™ (COS-P), a group-based attachment-informed preventative parenting education program. COS-P was chosen as the comparison intervention because it is currently delivered as the parenting treatment of choice in many government and community-based settings across Australia [49] and is of a similar treatment length (8 weeks). It is acknowledged that COS-P is not a perfect comparator as PCIT-T is delivered individually while COS-P is delivered to groups of 6–8 participants. To address this, the treatments will be matched for treatment length (8 weeks) and total treatment time (16 h), and study results will be interpreted with the different delivery modalities in mind.

We expect that of the two interventions, PCIT-T will be associated with the greatest treatment gains for the following reasons: (i) PCIT-T is the more intensive treatment model (PCIT-T is delivered on an individual basis; COS-P is a group program), (ii) PCIT-T is targeted at a narrower age range and is therefore more developmentally tailored (PCIT-T is for children aged 12–24 months; COS-P does not specify a child age range), (iii) PCIT-T is designed specifically for children with clinically significant disruptive behaviors and COS-P is not, and (iii) compared to COS-P, PCIT-T has an explicit behavioral component, which aligns with meta-analytic findings indicating that interventions that are brief, have a behavioral component, and which enhance parental sensitivity are most likely to bring about lasting improvements in children’s attachment security and thus capacity for behavioral and emotional regulation [50].

We thus hypothesize that from baseline (‘Time 1’) to post-treatment/waitlist (‘Time 2’) there will be a gradient effect, whereby parent-child dyads who receive PCIT-T will show the greatest gains and parent-child dyads who receive no treatment will show the least gains with respect to the following variables:

1. Improved parenting capacity through increased i) labeled praise of the child, ii) parenting sensitivity, iii) parent sense of competence in managing negative toddler emotions, iv) parent mentalizing about the child, and v) parent emotion regulation; and decreased i) negative statements directed toward the child, ii) parent sense of caregiving helplessness, iii) child abuse potential, and iv) parental stress.
2. Improved child outcomes including increased: i) emotion regulation maturity; ii) social-emotional functioning including initiative, relationship functioning and self-regulation, iii) attachment security, and iv) decreased externalizing behavior.

Second, we expect that for the two active treatment conditions, this gradient effect will be maintained at a 4-month post-treatment follow-up, whereby parent-child dyads that receive PCIT-T will show superior outcomes on all variables compared to parent-child dyads where the parent received COS-P.

## Methods

### Design

This study will be a RCT comparing outcomes for participants receiving one of two treatment conditions (PCIT-T, COS-P) and those receiving no treatment (waitlist condition).

### Participants and setting

Participants will be 90 toddlers aged 14–24 months with behavioral difficulties referred by a health professional (e.g., General Practitioner (GP), Paediatrician, Early Childhood Nurse, Psychologist) to the Karitane Toddler Clinic (KTC), a specialized outpatient child treatment clinic located in South Western Sydney, Australia. A decision was made to include children aged 14 months and over, rather than the 12 months and over that the PCIT-T intervention is designed for, to increase the likelihood that children in the sample are of a similar developmental stage (e.g., walking, starting to talk) at the time of participation. The study will be advertised using posters and flyers placed in the waiting rooms of GP and Early Childhood Clinics, and online (e.g. advertisements on parenting websites, Facebook and other social media platforms).

### Procedure

#### Recruitment

After being referred to the KTC, families will be informed about the research study and will undergo a clinical intake procedure including (i) an initial phone-based intake interview conducted by an Intake Officer, (ii) a 20-min in-depth phone-based intake assessment conducted by a member of the clinical research team, and, (iii) for complex cases, case review at a weekly multidisciplinary team meeting. A member of the clinical research team will then phone the families who appear suitable for the KTC service to schedule an initial face-to-face clinical assessment session. To be included in the study, the family must have a child aged 14–24 months, a referral from a health professional, and the parent must give a positive response to one or both of two screening questions (‘Do you have concerns about your child’s behavior?’ and/or ‘Do you have difficulties managing your child’s behavior?’). No other screening questions or measures will be used to assess eligibility, however families will be excluded from the study if there is evidence (in the information obtained in the referral or intake assessment) of severe parental depression with suicidality or other serious mental health conditions causing significant impairment in cognition or behaviors (e.g., psychosis) or if they are not sufficiently proficient in speaking English to complete study measures and protocols. Parents will be told that the study seeks to test different approaches to helping parents who are struggling with challenging toddler behaviour. Eligible and willing parents will provide written, informed consent to participate in the research during the first face-to-face assessment session at the KTC.

#### Randomization

**Fig. 1:**
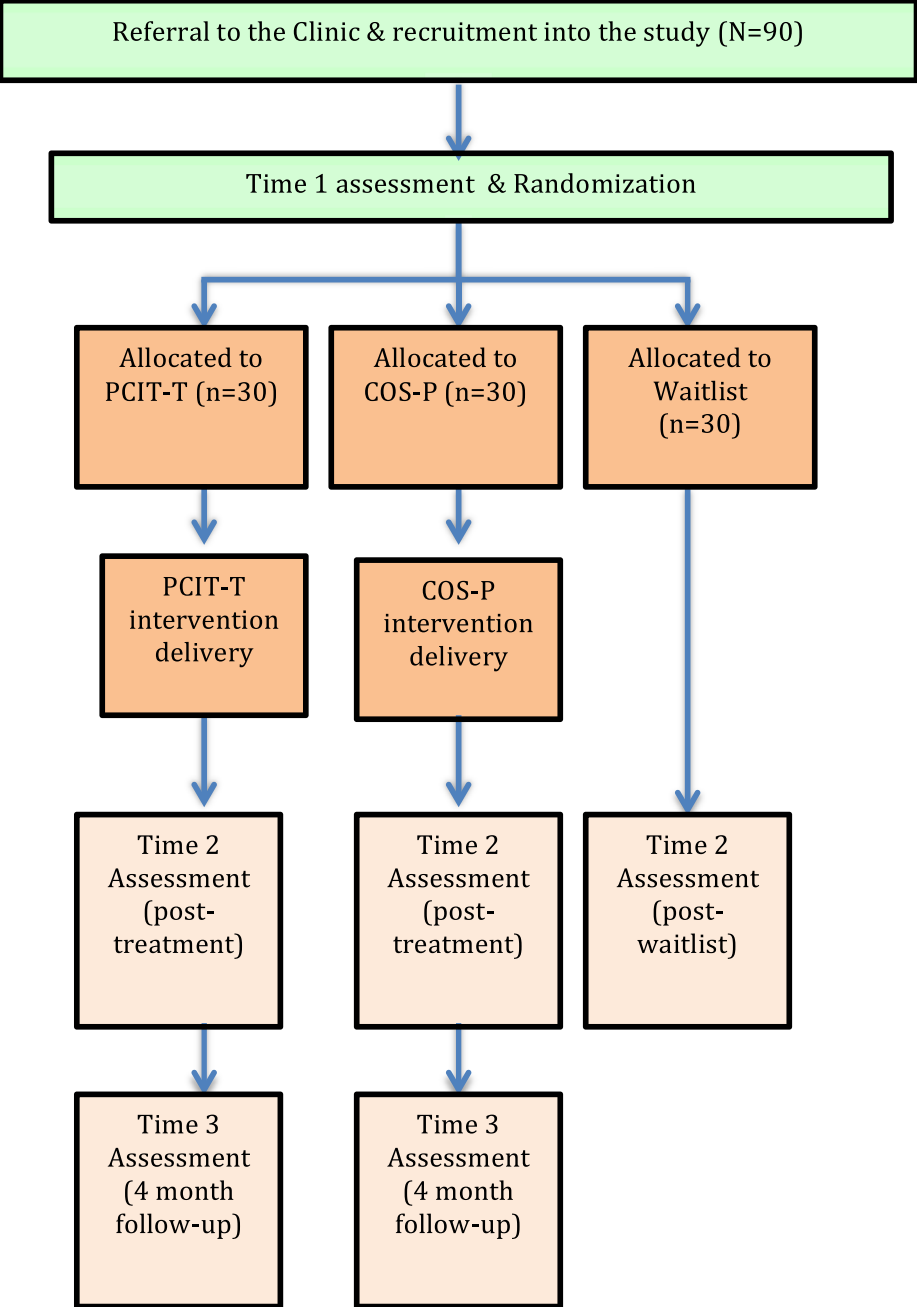
Participant flow through the study

Participants who meet the study inclusion criteria will be randomly assigned to receive one of the three conditions using restricted block randomization, using block sizes of *n* = 6. This means that within each block of *n* = 6, 2 participants will be allocated to the PCIT-T condition, 2 participants will be allocated to the COS-P condition and 2 participants will be allocated to the Waitlist condition. The order will be randomized within each block. Figure 1 shows the way that participants will flow through the three assessment points for the study (Time 1, T1; Time 2, T2; Time 3; T3). Randomization will occur at the start of the first face-to-face assessment session (T1), with allocations pulled out of pre-prepared sealed envelopes, but the research team and family will not be informed of the condition to which they have been allocated until after completion of the entire T1 assessment.

#### Time 1 assessment

All participants will complete the T1 assessment, conducted over three separate sessions in a one-week period. The first T1 session will be clinic-based and will include a clinical interview (family background, presenting problems, risk assessment) followed by three video-taped observational tasks. The first observational task will be the SSP [15], an experimental procedure comprising eight three-minute intervals involving separations and reunions between the mother and the child. The SSP will be later coded for child attachment pattern (see Measures section). The second observational task will be a structured parent-child interaction involving five sequential 4-min episodes (‘free play,’ ‘child-led play,’ a ‘frustration situation’ (toys taken out of the room), ‘toy reunion’ (toys returned to the room), and ‘clean up’), each preceded by standardised instructions provided to the parent from behind a one-way mirror through a wireless headset/bug-in-the-ear microphone. The parent-child interaction will later be coded for parent behaviour and sensitivity (see Measures section). The third observational task will be a ‘child frustration’ task [51] in which a large, locked Perspex box containing an unreachable colourful toy with flashing lights will be positioned in the centre of the room. The child will be left for 5 min to interact with the locked box while the parent completes a pen-and-paper questionnaire in the same room. Parents will be told to respond to the child’s advances as though they were needing to complete an urgent task before rejoining their child. The ‘child frustration’ task will later be coded for child emotion regulation maturity (see Measures section). The second T1 assessment session, also clinic based, will comprise the remainder of the clinical interview if not finished at the first session. The third T1 assessment session will be a 60–90-min home visit in which a research assistant will observe and video-tape the parent and child interacting in their natural environment in a series of semi-structured tasks including free play (with and without toys), reading a book, two divided attention tasks (parent completes a questionnaire and parent speaks with the researcher), a snack/mealtime, and playing a game with the researcher. The video-taped home visit will be later coded for infant attachment security (see Measures section). During the home visit, the parent will also be asked to provide a speech sample in which the parent will answer an open ended question, “What is your child like?” The speech sample will be audio-taped and transcribed verbatim, and then coded for parent mentalizing about the child, operationalized as mind-mindedness (see Measures section).

A series of parent-report questionnaires will be provided to the parent at the first T1 assessment session to be completed at home and returned during the T2 assessment session. For those unable to complete the questionnaires in this time frame, additional opportunity to complete questionnaires is provided during the home visit if required.

#### T2 and T3 assessments

Following the completion of the T1 assessment, participants will commence the treatment condition to which they have been allocated (details of interventions provided below). Participants allocated to an active intervention group (PCIT-T or COS-P) will commence treatment as soon as possible, after which they will complete the T2 assessment (immediately post-treatment, i.e., 8 weeks after the T1 assessment), and the T3 assessment (4-months after the T2 assessment). Participants allocated to the Waitlist group will complete the T2 assessment at the end of the 8-week waiting period. The T2 and T3 assessments will be identical to that of the T1 assessment, except that the SSP will not be repeated at T2.

#### Participant reimbursement

As compensation for time and travel expenses, participants will be offered a $25 gift voucher and a small gift of appreciation for the child after completion of each of the assessments.

See Fig. 1 for a summary of participant flow through the study.

### Interventions

#### Parent-child interaction therapy – toddler (PCIT-T)

PCIT-T will be delivered according to the protocol outlined by Girard and colleagues [42]. This will include direct live coaching (from behind a one-way mirror using a ‘bug-in-the-ear’) during parent-child play sessions (CDI-T and PDI-T phases) at the KTC. Families will attend a CDI-T teaching session followed by 6–8 CDI-T coaching sessions and then a PDI-T teaching session followed by 2–4 PDI-T coaching sessions. All families in this condition will receive a total of 16 h of therapy. This will include 1–2 individual parent or parent-child sessions over an 8-week period (equating to two 45-min sessions most weeks and allowing for some weeks with one 45-min session). The PCIT-T treatments will be provided on-site at the KTC and delivered by experienced clinicians who have been trained and accredited in the intervention. Treatment fidelity will be maximized by the use of (i) post-session fidelity checklists, completed by PCIT-T clinicians at the end of every treatment session [42] and (ii) clinical reflective supervision sessions provided to each of the PCIT-T clinicians on a fortnightly basis throughout the course of the research trial by one of the PCIT-T program developers.

Circle of Security – Parenting™ (COS-P) will be delivered according to the protocol outlined by Cooper, Hoffman, and Powell [49]. COS-P is a manualized eight-session parent-education group program; it has the same broad aims and core components as the COS-Intensive model from which it was developed (i.e., to increase caregiver sensitivity and responsiveness to child cues, empathy for the child by supporting parental reflective functioning, recognition and understanding of child attachment cues, and awareness of the impact of the caregiver’s own attachment history on caregiving patterns). The program is facilitator led, and uses archived clinical DVD footage of problematic parent-child interaction and healthy alternatives to illustrate attachment patterns and parenting styles, and promote group discussion. All parents in this condition will receive a total of 16 h of therapy. This will include eight, 2-h, parent-only group sessions (childcare provided). The COS-P groups will be provided on-site at the KTC, co-facilitated by two clinicians who have been trained and accredited in the intervention, at least one of whom has experience in running previous groups and who has no training or experience delivering PCIT-T. In total, approximately 6 different therapists will be involved as facilitators for the COS-P groups. Treatment fidelity will be maximized by the use of (i) fidelity checklists, completed individually by each of the group facilitators at the end of each session, and (ii) 1-h clinical reflective supervision sessions, provided to the group facilitators twice during the course of each group program by an experienced COS-P facilitator and clinical supervisor.

#### Waitlist

Participants allocated to this condition will complete the T1 assessment but will receive no treatment for the following 8-week period. They will then complete the T2 assessment, and this will represent the completion of their participation in the study. Families allocated to the waitlist condition will be offered the opportunity to receive PCIT-T or COS-P (whichever they choose) at the completion of the T2 assessment but this will not be for the research trial.

### Measures

For a summary of study measures, see Table 1. For all observational measures, coding will be conducted by a primary coder and 25% of cases double-coded by a second coder to ensure inter-rater reliability. All coders will be masked to treatment condition and assessment time point.

**Table 1:** Summary of key study variables and measurement tools, and assessment time points administered

Variable	Measure	Time 1	Time 2	Time 3^a^
*Parenting capacity outcomes*				
Positive parenting	Dyadic Parent-Child Interaction Coding System – Labeled Praise	✓	✓	✓
Negative parenting	Dyadic Parent-Child Interaction Coding System – Negative Talk	✓	✓	✓
Parenting sensitivity	NICHD Study of Early Child Care and Youth Development: Sensitivity Scales	✓	✓	✓
Sense of competence in managing negative toddler emotions	Coping with Toddler’s Negative Emotions Scale (CTNES)	✓	✓	✓
Caregiving helplessness	Care-Giving Helplessness Questionnaire (CHQ) - ‘mother helpless’ subscale	✓	✓	✓
Risk of abuse	Brief Child Abuse Potential Inventory (BCAP)	✓	✓	✓
Mentalizing about the child	Mind-mindedness rating system	✓	✓	✓
Emotion regulation	Difficulties in Emotion Regulation Scale (DERS)	✓	✓	✓
Parenting stress	Parenting Stress Index–Short Form (PSI-SF)	✓	✓	✓
*Child outcomes*				
Social-emotional functioning	Devereux Early Childhood Assessment (DECA)	✓	✓	✓
Child emotion regulation maturity	Observational 5-min ‘child frustration task’	✓	✓	✓
Attachment security	Attachment Q-sort (AQS)	✓	✓	✓
	Strange Situation Procedure (SSP)	✓	✗	✓
Child externalizing behaviour	Child Behavior Checklist (CBCL/1.5–5)	✓	✓	✓

^a^ Time 3 measures only administered to participants in the PCIT-T and COS-P groups

*Positive and negative parenting* during the 20-min parent-child interaction will be coded according to the Dyadic Parent-Child Interaction Coding System, Fourth Edition, a coding scheme designed to rate parent-child verbal interactions and responses during observed interactions [52]. The DPCIS has been shown to have high levels of reliability, with kappa coefficient scores typically over .80 [53, 54]. Trained DPICS coders will view the video-recorded parent-child interaction sessions, create a moment-by-moment transcript of the parent-child interactions, and use the transcription and video to code every parent verbalization. A ‘positive parenting’ score (total number of labeled praises) and a ‘negative parenting’ score (total number of negative statements) will be calculated based on the number of times these verbalisations are observed during the five structured situations. To create standardized scores, frequency counts will be transformed into ratios based on the following: 1) total number of labelled praises divided by total number of verbalisations, and 2) total number of negative statements divided by total number of verbalizations.

*Parenting sensitivity* during each episode of the 20-min parent-child interaction will be coded using the NICHD Study of Early Child Care and Youth Development: Sensitivity Scales (NICHD-SECCYD) [55]. This coding system has demonstrated reliability (intra-class coefficents typically .8 and over [56, 57]) and validity (positive associations with infant attachment quality [56–58], and sensitivity to change following parenting interventions [59]). The NICHD-SECCYD sensitivity scales yields a composite parental sensitivity score, calculated by summing scores on six scales, each scored on a 4-point scale whereby higher scores indicate that the variable is more characteristic of the parent: sensitivity, positive and negative (reversed) regard for the child, intrusiveness, respect for autonomy and hostility (reversed).

*Parental sense of competence in managing negative toddler emotions* will be assessed using the Coping with Toddler’s Negative Emotions Scale (CTNES) [60]. The CTNES comprises 12 hypothetical scenarios in which the child becomes upset, angry, or distressed. The caregiver is asked to rate their likelihood of engaging in each of seven possible responses to their child’s negative emotions: (a) distress reactions, (b) punitive reactions, (c) minimizing reactions, (d) expressive encouragement, (e) emotion-focused reactions, (f) problem-focused reactions, and (g) granting the child’s wish. For each scenario, caregivers rate each possible response from 1 (very likely) to 7 (very unlikely). Previous studies have shown the CTNES to have a high level of internal consistency (alphas for individual subscales ranging from .68–.93) and test-re-test reliability (*r*s = .65 to .81) [60].

*Parent sense of caregiving helplessness* will be assessed using the Caregiving Helplessness Questionnaire (CHQ) [61] (‘mother helpless’ subscale). The scale comprises 7 items, each answered on a five-point scale, with higher scores indicating greater helplessness. The helplessness subscale of the CHQ has been shown to possess good construct validity and to be internally consistent (αs .80–.85) [44, 62].

*Parent mentalizing about the child, operationalized as mind-mindedness,* will be coded from the speech sample question (“What is your child like?”), obtained during the home visit. Coding will be conducted according to Meins and Fernyhough’s mind-mindedness coding guidelines [63]. Participant responses will be transcribed verbatim from audio-tape and then the primary coder will section the transcription into individual comments, based on temporal (1-s gap) or semantic discontinuities. Each individual comment will then be coded as belonging to one of five categories, namely ‘Mental’, ‘Behavioral’, ‘Physical’, ‘General’, ‘Self-referential’). Comments coded as ‘Mental’ attributes will be descriptors related to the child’s mental life, intentions, interests, likes and emotions. Behavioral attributes will be comments that refer to the child’s behavior (e.g., games or activities that the child does). Physical attributes will be comments about the child’s appearance, position in the family or age, and general attributes will include comments that don’t fit into the other three categories. The self-referential code will be given when the parent speaks about themselves, rather than the child. A ‘mental state’ frequency score will be calculated as the frequency count of ‘mental’ comments as a proportion of the total number of verbalisations, to control for verbosity. Meins’ coding system for mind-mindedness has been well validated, with previous studies showing good inter-rater reliability and stability over time (e.g., McMahon and colleagues reported intraclass correlations for individual categories ranging from .89–.92 and showed that mothers who made more mind-related comments at 7 months postpartum, also did at 19 months) [64]. Construct validity has also been demonstrated with higher mind-mindedness scores having been shown to predict maternal sensitivity [65] and infant attachment [27].

*Parental emotion regulation* will be assessed using the Difficulties in Emotion Regulation Scale (DERS) [66], a 36-item, self-report measure of emotion dysregulation. The DERS yields a total score (range 36–80, higher scores indicating greater emotion dysregulation) and six subscale scores: (1) Nonacceptance of emotional responses, (2) Difficulties engaging in goal directed behavior, (3) Impulse control difficulties, (4) Lack of emotional awareness, (5) Limited access to emotion regulation strategies, and (6) Lack of emotional clarity. The DERS has been shown to have high internal consistency (α = .93), good-test retest reliably (intra-class correlation coefficient = .88), and adequate construct and predictive validity [66].

*Parenting stress* will be assessed using the Parenting Stress Index–Short Form (PSI-SF), a 36-item self-report measure of stress in the parent–child system [67]. The PSI-SF comprises three subscales: ‘Parental Distress’, ‘Parent–child Dysfunctional Interaction’, and ‘Difficult Child’, with higher scores indicating greater dysfunction. The internal consistency (α = .71–.92), test re-test reliability α = .06–.80), and convergent validity of the PSI-SF subscales and the total score have been demonstrated in a similar sample of English-speaking parents of young toddlers with behavioral problems [68].

*Child abuse potential* will be assessed using the Brief Child Abuse Potential Inventory (BCAP) [69]. The BCAP is a parent-report scale that comprises 24-items, each rated as ‘agree’ or ‘disagree’. The BCAP is an abbreviated version of the widely-used 160-item Child Abuse Potential Inventory [70]. Internal consistency (α = .89) and construct validity have been demonstrated [69].

*Child social-emotional functioning* will be assessed using the Devereux Early Childhood Assessment (DECA) [71, 72], a strengths-based parent-report scale designed to assess emotional, social, and behavioral capacities during early childhood. There are two aged-based versions of the DECA, an infant version (DECA-I; designed for ages 1–18 months; 33 items) and a toddler version (DECA-T; designed for ages 18–36 months; 36 items). The DECA-I comprises two scales (initiative, attachment/relationships) and a Total Protective Factors scale, which is a composite of the initiative and attachment/relationships scales. The DECA-T comprises three subscales (initiative, attachment/relationships, self-regulation) and the Total Protective Factors composite. In this study, participating children will complete the DECA-I or DECA-T depending on their age. The DECA-I and DECA-T subscales, rated by parents, have been shown to be internally consistent (total protective factors α = .90–.94), to show good test-retest reliability (*r*s = .71–.99) [72].

*Toddler emotion-regulation maturity* will be rated from videotapes of the 5-min ‘toddler frustration task’ using the coding protocol developed by Johnson [51]. This will involve first, the level of distress shown by the toddler being coded in 30 × 10 s intervals, on a four-point scale incorporating both intensity and duration of distress, with higher scores indicating a higher level of distress. Scores will be summed to yield a total toddler distress score (possible range 0–90). Second, regulatory behaviors articulated by Calkins et al., [73] (attentional deployment, situation modification and cognitive change) will be coded on a four-point scale during the same time intervals. Based on Gross’ (2007) theoretical framework for the developmental hierarchy of emotion-regulation strategies [74], these regulatory behavior scores will be arranged hierarchically into three scales reflecting least mature (attentional deployment) to most mature (cognitive change) regulatory strategies and summed to yield a composite emotion-regulation maturity score. Maternal support seeking will also be coded in the same way as regulatory behaviors and will be examined as a separate scale.

*Child attachment security* will be assessed in two ways so that both dimensional and categorical ratings can be obtained. First, to examine attachment security dimensionally, the video-taped home visit will be coded using the Attachment Q-sort (AQS) [75, 76], a validated coding procedure designed to assess attachment security in a naturalistic environment in children aged 12–48 months. In the AQS, various descriptors of child behavior, including attachment behavior, are sorted into nine piles from “most characteristic” to “least characteristic.” An overall security score is derived by correlating the sort for each child with a criterion sort of the prototypical behavior of a secure child. Second, to examine attachment security categorically, including disorganization, child behavior observed during the SSP will be coded according to the coding systems of Ainsworth [15] and Main and Solomon [17]. The SSP has demonstrated validity [13, 77] and is widely considered to be the gold standard measure of infant attachment. The SSP comprises eight three-minute episodes involving separations and reunions between the mother, the child and a friendly stranger. Infants are subsequently assigned to one of three ‘organized’ classifications based on their observed behavior, namely secure (B), anxious-avoidant (A), or anxious-resistant (C), following the Ainsworth coding system [15]. In addition, a primary ‘disorganized’ (‘D’) classification can also be applied with the ABC classifications as secondary, using procedures described by Main and Solomon [17]. SSP coding will be conducted by coders who have undertaken a 10-day training and been certified as a reliable coder by attaining at least 80% agreement with a SSP trainer.

Parent-reported *externalizing child behavior* will be assessed using the Child Behavior Checklist for ages 1.5–5 years (CBCL/1.5–5) [78], a validated parent/teacher report scale designed to measure behavioral, emotional and social functioning in children aged 1.5–5 years, and which has been used reliably with children as young as 12 months [46, 79]. The CBCL/1.5–5 comprises 99 items, each rated in terms of the frequency with which the child displays given problem behaviors on a scale of 0–2 (higher scores indicating presence of the behavior). CBCL scores can be summed to yield a ‘total problems’ score and an externalizing sub-scale score can also be computed. In the current study, the parent report version of the CBCL/1.5–5 and the externalizing sub-scale score will be used.

### Statistical analysis

Continuous outcome variables will be analyzed across three time conditions (T1, T2, T3) using a linear mixed models repeated measures design. For all continuous variables, group differences will be tested with family-wise adjustments made to the raw *p*-values to account for multiple comparisons using Holm’s [80] stepdown Bonferroni procedure. Group by time interactions will be examined in fixed effects models, with planned comparisons constructed to examine the differences between the three groups at T2, and between the PCIT-T and COS-P groups at T3. Intention-to-treat analyses (ITT) [81] will be used, with participants included in the analyses within their randomly assigned treatment condition regardless of the amount of treatment received and group mean values estimated based on all observed data of those in the ITT sample. The clinical significance of differences on continuous study variables (from T1 to T2) will be assessed using Cohen’s *d* with effect sizes evaluated using Cohen’s [82] guidelines. Reliable change index scores will also be calculated to assess whether the magnitude of individual-level change on continuous study measures exceeds the margin of measurement error [83]. To examine changes in categorical SSP classifications from T1 to T3 (SSP not administered at T2), generalized linear mixed models with binomial distribution and logit link will be conducted with insecure attachment (0/1) and disorganized attachment (0/1) as dependent variables.

### Sample size and power calculations

Required sample size was calculated to detect a group by time interaction of moderate size (*f* = .21), for 3 groups and 2 time points (T1 and T2). The effect size *f* is defined as *f* = *σ*_*m*_*/σ*, where *σ*_*m*_ is the standard deviation (*SD*) of the effect of interest and *σ* is the common error *SD*. Cohen (1963) suggested that *f* of .25 is a moderate effect. This effect size was chosen because (a) it is a reasonable and achievable statistical effect, and (b) it is substantively and clinically meaningful for the primary outcome measures. The CBCL externalizing subscale score was used in the power calculation because of all the measures in the study (a) it is the one that corresponds most directly to the presenting problem for most participants; and (b) it was used in a previous study conducted at the same site and with a similar study sample, allowing us to obtain realistic estimates of mean CBCL externalizing subscale scores for T1, and for the PCIT-T group at T2. Based on our previous research using a similar sample and the CBCL externalizing subscale as an outcome measure [48], at T1, all groups were assumed to have a mean of 24. Means at T2 were assumed to be 13 (PCIT-T), 16 (COS-P) and 22 (Waitlist). A common SD of 9 and a correlation between measurements of .6 were specified and it was calculated that assuming alpha of .05 and 80% power, a minimum total of 48 participants (i.e. *n* = 16 in each group) would be required. Post hoc testing of differences between the three groups at T2, and a PCIT-T versus COS-P difference at T3, show that *n* = 30 in each group would give 80% power to detect a mean difference between groups of at least 8.0, with *SD* of 9. And alpha of .05/4, to correct for multiple comparisons for all group comparisons at T2 (PCIT-T versus COS-P, PCIT-T versus Waitlist, COS-P versus Waitlist) and T3 (PCIT-T versus COS-P).

## Discussion

Evidence points to the value of parent-child relationship focused interventions for children with early onset attachment and/or behavioral difficulties. Significantly, evidence suggests that interventions that are brief, have a behavioral component, and enhance parental sensitivity are most likely to bring about lasting improvements in children’s attachment security and thus capacity for behavioral and emotional regulation [50]. PCIT-T is a new, innovative early parenting intervention that aligns with these recommendations and is designed to treat families with toddlers who are displaying persistent and significant disruptive behavior difficulties. Like standard PCIT, the program is centered around dyadic parent-child play sessions, with the therapist providing live, in-the-moment feedback and support. PCIT-T aims to increase positive parenting skills (e.g. praise) and sensitivity and decrease use of negative behaviors (e.g. criticism) but there is also a focus on helping parents to better manage their emotions so that they are better placed to help their children manage theirs.

The design of this study has limitations including the different mode of delivery for the two interventions (PCIT-T is delivered individually, COS-P is delivered in a group), the large number of study measures utilized which places burden on participants, and the fact that the waitlist control group will not take part in the 4-month follow-up due to ethical reasons associated with denying patient care for this extended period of time. Finally, while the parent-report measure of child externalizing problems, the CBCL, has been used successfully in previous studies with children aged as young as 12 months [79], it has not been validated for children aged less than 18 months and so there may be issues with the measure for children in the study aged 14–17 months. Despite these limitations, however, the study has a number of strengths including the RCT design, utilization of a clinical sample and a comprehensive mix of parent-report and observational measures.

PCIT-T, delivered in the early toddlerhood period when there is greater plasticity of the developing brain, when capacities for emotional and behavioral control are developing, and when internal representations of attachment relationships are in the critical stages of formation, has the potential to bring about significant and lasting changes for children and families. The current study will evaluate the effectiveness of the most recent iteration of the PCIT-T model [42] and will build on previous pilot studies [45–48] to add to current knowledge about the effectiveness of this new intervention model.

## Data Availability

Not applicable.

## Abbreviations

ABC: Attachment and Biobehavioral Catchup
AQS: Attachment Q-Sort
BCAP: Brief Child Abuse Potential Inventory
CBCL/1.5–5: Child Behavior Checklist for ages 1.5–5 years
COS-I: Circle of Security Intensive Intervention
COS-P: Circle of Security – Parenting™
CDI-T: Child Directed Interaction – Toddlers
CHQ: Caregiving Helplessness Questionnaire
CTNES: Coping with Toddler’s Negative Emotions Scale
DECA: Devereux Early Childhood Assessment
DECA-I: Devereux Early Childhood Assessment - infant version
DECA-T: Devereux Early Childhood Assessment - toddler version
DERS: Difficulties in Emotion Regulation Scale
DPCIS-IV: Dyadic Parent-Child Interaction Coding System, Fourth Edition
GP: General Practitioner
ITT: Intention-to-treat analyses
KTC: Karitane Toddler Clinic
NICHD-SECCYD: NICHD Study of Early Child Care and Youth Development: Sensitivity Scales
PCIT: Parent Child Interaction Therapy
PCIT-T: Parent Child Interaction Therapy – Toddler
PDI-T: Parent Directed Interaction-Toddlers
PSI-SF: Parenting Stress Index–Short Form
SSP: Strange Situation Procedure
RCT: Randomized Controlled Trial
T1: Time 1
T2: Time 2
T3: Time 3
VIPP-SD: Video-feedback Intervention to promote Positive Parenting and Sensitive Discipline
